# Brain volumes are related with motor skills at late childhood in children born extremely preterm

**DOI:** 10.1371/journal.pone.0326041

**Published:** 2025-06-13

**Authors:** Lina Broström, Hedvig Kvanta, Maria Örtqvist, Nelly Padilla, Ulrika Ådén

**Affiliations:** 1 Department of Clinical Science and Education, Karolinska Institutet, Stockholm, Sweden; 2 Sachs’ Children and Youth Hospital, Stockholm, Sweden; 3 Neonatal Research Unit, Department of Women’s and Children’s health, Karolinska Institutet, Stockholm, Sweden; 4 Functional Area Occupational Therapy & Physiotherapy, Allied Health Professionals Function, Karolinska University Hospital, Stockholm, Sweden; 5 Department of Biomedical and Clinical Sciences, Linköping University, Linköping, Sweden; 6 Neonatal Unit Karolinska University Hospital, Stockholm, Sweden; Massachusetts General Hospital, UNITED STATES OF AMERICA

## Abstract

**Background:**

This study had three aims. First, we wanted to explore if there was difference in motor performance at 12 years of age in children born extremely preterm (EPT < 28 weeks of gestation) and at term. Our second aim was to study whether the volumes of motor networks and regions differed between those groups when they underwent brain scans at 10 years of age. Third, we investigated whether there were differences in the motor networks and regions of the brain in children born EPT who did or did not have motor impairment at 12 years of age.

**Methods:**

In a Swedish national study, a subgroup of 42 children born before 27 weeks and 25 term-born controls underwent MRI at age 10. A neuroradiologist performed MRI acquisitions, and analyses focused on brain regions associated with motor function. At age 12, motor function was assessed using the Movement Assessment Battery for Children – Second Edition (MABC-2), conducted by a licensed physiotherapist. Examiners were blinded to group status. Motor function and motor-related brain volumes were compared between the EPT and control group, and between children born EPT with and without motor impairments.

**Results:**

Findings revealed significantly reduced motor performance and smaller motor region volumes in EPT children compared to controls (p < 0.001). Among EPT children, those with motor impairment especially in aiming and catching, had notably smaller brain volume in the basal ganglia (mean difference:1.2 cm^3^, p = 0.049), cerebellum (mean difference:14.4 cm^3^, p < 0.001), motor execution (mean difference:3.7 cm^3^, p = 0.049) network and motor imagery network (mean difference 5.6 cm^3^, p = 0.049) than their EPT peers without such impairments. Cerebellar volume remained significant different between the groups when adjusting for birth weight and sex in a linear regression model, p = 0.02 (η^2^ = 0.17).

**Conclusion:**

The results underscore the impact of extreme prematurity on motor function and brain structure, highlighting a specific link between reduced motor area volumes and impaired ball skills.

## Introduction

Development in perinatal and neonatal intensive care has been associated with increased rates of survival among children born extremely preterm (EPT < 28 weeks of gestation). However, many of these children develop neurodevelopmental impairment (i.e., cognition, language, motor problems) [1]. Serenius et al reported that 20% of 441 children born EPT had moderate disabilities at 6.5 years of age, 13% had severe disabilities and 67% had no or mild disabilities [2]. Children born EPT have more motor impairments than term-born children and it is well known that these include aiming and catching, balance and fine motor skills [3]. A meta-analysis of motor development in children born very preterm or with very low birth weight, from infancy to adolescence, showed they had significantly worse motor performance than term-born children in Movement Assessment Battery for Children (d = −0.65 (95% CI, −0.70 to −0.60; P < .001) [4].

EPT births occur during a vulnerable time of brain development. Disturbances in the maturation of the brain, including altered myelination, synaptic development and cortical organization lead to different brain development than term-born children [5,6]. Between 24 and 40 weeks of gestation the cerebellum increases five fold in volume. The underdevelopment of cerebellum, with loss of volume in children born EPT can be caused by different mechanisms including inset of hemosiderin, hypoxia and reduced excitatory input leading to atrophy [7]. The development of grey matter can be disturbed by diffuse white matter injury, intraventricular haemorrhage (IVH) or independently, including inflammation and hypoxia. Dysmaturation, loss of function of dendrites and loss of interneurons have been described causing signal disturbance and dysmaturation of the folding of the cortex [8].

The use of advanced magnetic resonance imaging (MRI) techniques has made it possible to study alterations in the global and local cerebral development of preterm infants. Volumetric measurements performed at term age and through adolescensens have also shown global and regional differences in brain volumes between children born EPT and at term [9–11]. A study by Dewey et al showed that children who were born very preterm, and had later motor impairment, had smaller brain volumes at term age and at seven years of age than children born very preterm with normal motor function [12].

However, there is increasing awareness that prematurity is characterized by widespread abnormalities throughout the brain and that these involve neural networks. We are not aware of any studies that have compared the relationship between motor networks in the brain and motor performance in children born EPT and at term when they reach late childhood.

This study had three aims. First, we wanted to explore if there was difference in motor performance at 12 years of age in children born EPT and at term. Our second aim was to study whether the volumes of motor networks and regions differed between those groups when they underwent brain scans at 10 years of age. Third, we investigated whether there were differences in the motor networks and regions of the brain in children born EPT who did or did not have motor impairment at 12 years of age.

We had three hypotheses. First, children born EPT would have worse motor performance at 12 years than term-born peers. Second, that the areas of the brain involved in motor function would be smaller at 10 years of age in children born EPT than children born at term. Third, that children who were born EPT and had motor impairment would have smaller brain volumes in the networks and regions involved in motor performance, than those EPT without motor impairment at 12 years of age.

## Materials and methods

### Participants

**Fig 1:**
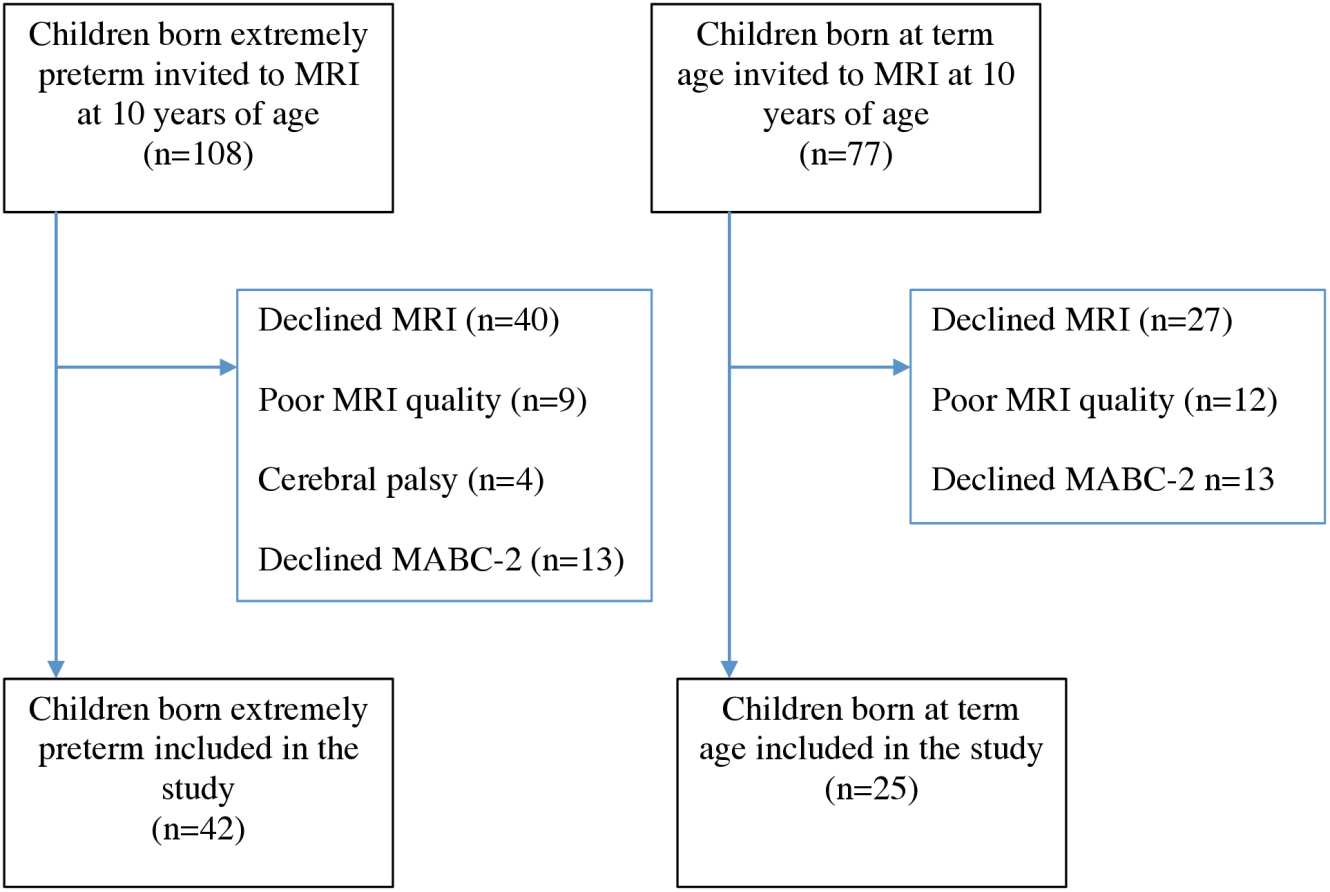
Flow-chart of the study population.

We invited 108 children born EPT in Stockholm, Sweden to participate in this national study. These participants constitute a regional subgroup of the national EXPRESS cohort (Extremely Preterm Infants in Sweden Study). They were all born before 27 weeks of gestation, between 1 January 2004 and 31 March 2007 (Fig 1). Of these, 107/108 had undergone an MRI brain scan at term age. Cranial ultrasounds were performed during the neonatal period and any cases of IVH and periventricular leukomalacia were diagnosed. At 10 years of age, the children were invited to undergo a MRI of the brain between 01/06/2015 and 18/04/2016. At 12 years of age between the years 03/06/2016 and 09/10/2019 their motor performance was examined using the Movement Assessment Battery for Children – Second Edition (MABC-2) [13]. The Swedish Medical Birth Registry was used to identify 77 term-born children who were matched to the EPT cohort by age, postcode and country of origin. They formed the control group and were also invited to undergo the same MRI scans and motor performance assessments at 10 and 12 years of age, respectively. Children born EPT, who were diagnosed with cerebral palsy, were excluded from the study. The regional ethics committee in Stockholm approved the study and written, informed parental consent was obtained for all participants.

### Background characteristics

Background descriptive data were collected from the Swedish Medical Birth Registry. Variables included gestational age, birth weight, sex and other relevant variables included in Table 1. Education level from the mother and age at assessment were collected at the follow up at 12 years in a questionnaire. Socioeconomic status was estimated using maternal education level, categorized as higher if they had university education. These variables were examined as potential covariates in the statistical analyses. Specifically, birth weight and sex were included as covariates in the group comparisons.

**Table 1:** Characteristics and magnetic resonance imaging findings for the whole study population.

	Preterm^a^ (n = 42)	Term (n = 25)
**Perinatal**		
Birth weight (grams), mean ± SD	835 ± 151	3717 ± 435
Gestational age at birth, weeks, median (range)	25.6 (23.1-26.6)	40.0 (38.0-41.6)
Male sex, n	21	12
Small for gestational age, n	3	0
Antenatal steroids, n	39	0
BPD, oxygen at age 36 weeks, n	14	0
Intraventricular haemorrhage I-II, n	14	0
Intraventricular haemorrhage III-IV, n	2	0
Mechanical ventilation (days), median (range)	6 (0-55)	0
Necrotizing enterocolitis Bell´s grade 2–3, n	7	0
Patent ductus arteriosus, treated with ibuprofen, n	26	0
Patent ductus arteriosus, surgical ligation, n	12	0
Retinopathy of prematurity, lase treatment, n	6	0
Sepsis, n	29	0
**Magnetic resonance imaging at term age**		
Normal white matter, n	23	25
Mild white matter abnormality, n	17	0
Moderate white matter abnormality, n	1	0
Gray matter abnormality, n	1	0
Cerebellar injury, hemosiderin/atrophy n	3/1	0
**Magnetic resonance imaging at 10 years**		
Discrete white matter abnormality, n	22	2
Cerebellar injury, n	0	0
Intracranial volume, mean ± SD	1380.3 ± 88.8	1442.8 ± 107.9
Age at scan, median (range)	9.9 (9.0-11.4)	10.2 (9.0-11.6)
**Sociodemographic data**		
Maternal education ≥ University level, n	28	14

Fisher’s exact test were used to identify differences in categorical data between the groups, the student’s *t*-test were used for continuous data were mean is presented and The Mann–Whitney U test were median is presented. Significant values defined as p < 0.05. SD, standard deviation; BPD, bronchopulmonary dysplasia. Sepsis defined as positive blood cultures or clinical picture of sepsis in association with elevated C-reactive protein or leukocyte count. ^a^One child born extremely preterm did not have MRI scans at term age.

### Outcome measures

The children’s motor performance was assessed using the MABC-2 [13], which is a standardized tool with high validity and reliability. It contains three domains: manual dexterity, aiming and catching and balance. Age-specific standard scores and percentiles are calculated from the raw scores of the three domains and these are added together to provide a total score. We analysed the total MABC-2 scores and domains and converted these into percentiles. This enabled us to identify the children born EPT who had motor impairment, which was defined as ≤5^th^ percentile according to the reference population in the manual [13].

### MRI acquisition

Neonatal MRI brain scans were performed at term age in all but one of the children born EPT. Details of the MRI scanning protocol have previously been published [14].

At 10 years of age, the children born EPT underwent MRI brain scans using a Sigma 3.0-T MR scanner (GE Healthcare, Illinois, USA) at the Karolinska University Hospital, Stockholm, Sweden. The MRI protocol included a sagittal 3D-T1 weighted with a BRAVO SPGR sequence: time to inversion was 400 ms, field of vision was 240 × 240 mm^2^; flip angle was 12^°^; voxel size was 1 × 0.938 × 0.938 mm^3^ and slice thickness was 1.0 mm.

### MRI processing and atlas-based segmentation

The assessment and scoring of the MRI images by a neuroradiologist have previously been described at term age^14^ and at 10 years of age [15].

The brain was segmented into 45 anatomical regions per hemisphere, according to an anatomic atlas, the AAL atlas [16]. The T1 weighted 3-dimension MRI images were pre-processed, as previously described including reorientation, removal of non-brain tissue and neck cropping [17]. The Linear Image Registration Tool FSL FLIRT (FMRIB, John Radcliffe Hospital, Oxford, UK) was used for AAL atlas co-registration to the native space of each child, using affine registration. A script written in MATLAB (MathWorks, Massachusetts, USA) was used to calculate the atlas volumes and is available to share upon request. The MRI images of each step were visually inspected.

Based on previous reports [18–21] we defined the motor execution network (precentral gyrus and postcentral gyrus) and the motor imagery network (precuneus, supplementary motor area, superior frontal gyrus, precentral gyrus). The volume of each brain region was determined by summing the volume of their components. We also included the thalamus, basal ganglia, and cerebellum, as they are part of the areas involved in the motor performance.

The whole structure of the cerebellum of each 3D MRI image was manually segmented with ITK-SNAP software [22]. Manual segmentation was performed due to deficiencies in automatic segmentation. Specifically, we observed that cerebellar boundaries were often poorly delineated or partially missing in a substantial number of cases, resulting in unreliable volume estimates (S1 Fig). These issues prompted us to perform manual segmentation to ensure anatomical accuracy and consistency across subjects. A quantitative evaluation of the segmentation was performed using the Dice Coefficient [23]. The agreement calculation was based on the segmentation of 10 subjects by two of the authors (LB, NP). The agreement was 0.94 ± 0.004, which exceeded the definition of excellent agreement (> 0.75).

### Statistical analysis

SPSS 26.0 (IBM Corp, New York, USA) was used for the statistical analysis. The chi-square test, Fisher’s exact test or the chi-square test for trends were used to identify differences in categorical data between the groups. The Mann–Whitney U test or the student’s *t*-test were used for continuous data. We compared children born EPT and at term in neurodevelopmental assessment and intracranial volume at 10 years and we also compared children born EPT with and without motor impairment. The general linear model was used to examine differences in motor networks and regions between the groups when the covariates were controlled for. Variables were included in the final regression models if there was evidence from a clinical perspective that they might be associated with the dependent variables, or they were variables that were significant in a univariate test with a p-value< 0.1 or significant between groups. Finally birth weight and sex was chosen as covariate also due to co-linearity. Because of the colinearity in certain variables of interest, a single variable was selected based on its strength of primary significance with the dependent variable. If the intracranial volume between two groups was significantly different we added it as a covariate. Intracranial volume was also correlating with birth weight, and because of colinearity between intracranial volume and birth weight we selected only one variable based on its strength of primary significance with the dependent variable. Assumptions about the requirements were met by the analyses. Statistical significance was a two-sided *p* value of less than 0.05.

## Results

### General characteristics

The study comprised 42 children born EPT and 25 children born at term (Fig 1). Six children born EPT were excluded from the cerebellum analysis, one due to a cyst and atrophy at term age and five due to poor image quality. The characteristics of the whole study group are presented in Table 1 and the characteristics of the children born EPT, with and without motor impairments, are shown in S1–S4 Tables. There were significant differences in birth weight and days on mechanical ventilation between the children with and without motor impairment in total motor and manual dexterity performance (S1 and S2 Tables).

### Motor assessment

There were significant differences in motor performance at 12 years of age between the EPT and term groups. Median total MABC-2 score was 71 (range: 32–99) in the EPT group compared with 83 (range: 59–96) in the control group (p < 0.001). Median score for children born EPT was 23 (8–39) and for term born children 29 (15–35) in manual dexterity (p = 0.001). Median scores in aiming and catching were 16 (8–31) for children born EPT and for term born children 19 (11–28), (p = 0.01). Median score in balance for children born EPT was 28 (8–36) and for term born children 35 (20–36), (p = 0.001, Table 2). The standard scores differed accordingly (Table 2).

**Table 2:** Results of MABC-2 for children born extremely preterm and term born when they reached 12 years of age.

MABC-2	Preterm (n = 42)	Term (n = 25)	P-value
Total test score, median (range)	71(32-99)	83(59-96)	**<0.001**
Total standard score, median (range)	8(2-16)	11(6-15)	**<0.001**
Manual dexterity component score, median (range)	23(8-39)	29(15-35)	**0.001**
Manual dexterity standard score, median (range)	8(2-15)	10(4-100)	**0.001**
Aiming and catching component score, median (range)	16(8-31)	19(11-28)	**0.01**
Aiming and catching standard score, median (range)	8(2-18)	10(4-15)	**0.02**
Balance component score, median (range)	28(8-36)	35(20-36)	**0.001**
Balance standard score, median (range)	9(1-14)	14(6-14)	**0.001**

Mann–Whitney U test were used for continuous data. Significant values was defined as p < 0.05.

### Brain volumes of motor networks

Comparing the brain volumes of the motor networks showed that the children born EPT had significantly smaller volumes. Mean of the motor execution network was 88.3 ± 5.7 in children born EPT and 94.3 ± 5.7 in children born at term, p < 0.001. In the motor imagery network mean was 142.2 ± 9.1 in children born EPT and 151.8 ± 9.2 in term-born children, p < 0.001. The basal ganglia, thalamus and cerebellum were also smaller in children born EPT with mean 27.9 ± 1.8, 12.9 ± 0.8, 130.8 ± 13.6 respectively, compared to the term-born group with mean 29.8 ± 1.8, 13.8 ± 0.8, 144.6 ± 11.3 respectively after correction for multiple comparisons and adjusted for intracranial volume (p < 0.001). The cerebellum was the most significantly affected area (Table 3).

**Table 3:** Comparison of the brain volumes of the motor networks in cm^3^ at 10 years between children born extremely preterm and term born.

Brain region mean ± standard deviation	Preterm (n = 42)	Term (n = 25)	P-value*	P-value Benjamin-Hochberg
Thalamus	12.9 ± 0.8	13.8 ± 0.8	**<0.001**	**<0.001**
Basal ganglia	27.9 ± 1.8	29.8 ± 1.8	**<0.001**	**<0.001**
Cerebellum	130.8 ± 13.6	144.6 ± 11.3	**0.001**	**0.001**
Motor execution network	88.3 ± 5.7	94.3 ± 5.7	**<0.001**	**<0.001**
Motor imagery network	142.2 ± 9.1	151.8 ± 9.2	**<0.001**	**<0.001**

*General linear model with intracranial volume as the covariate. Significant values defined as p < 0.05.

Notably, 13 of the EPT children had a Total MABC-2 score equal to or below the 5th percentile, aligning with one of the diagnostic criteria for developmental coordination disorder (DCD) [3].

**Fig 2:**
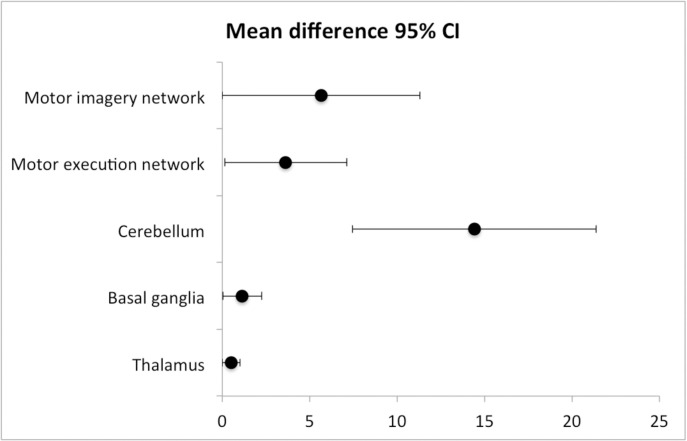
Comparisons of mean volume differences in cm^3^ in regions related to motor networks between children born extremely preterm who did, and did not, display motor impairment in the aiming and catching test.

Children born EPT with motor impairments in aiming and catching had significantly smaller volume in the basal ganglia with mean 27.1 ± 1.5 compared to children born EPT with normal aiming and catching with mean 28.3 ± 1.8, p = 0.049. Mean of motor execution network in children born EPT with motor impairments in aiming and catching was 85.8 ± 4.8 compared to children born EPT with normal aiming and catching with mean 89.5 ± 5.7, p = 0.049. Children born EPT with motor impairments in aiming and catching had significantly smaller volume in the motor imagery network with mean 138.3 ± 7.8 compared to children born EPT with normal aiming and catching with mean 143.9 ± 9.2, p = 0.049. Mean of the cerebellum in children born EPT with motor impairments in aiming and catching was 120.8 ± 6.9 compared to children born EPT with normal aiming and catching with mean 135.2 ± 13.6, p=<0.001 (Table 4 and Fig 2). Cerebellar volume remained significant different between the groups when adjusting for birth weight and sex in a linear regression model, p = 0.02 (η^2^ = 0.17).

**Table 4:** Comparison of brain volumes of motor networks in cm^3^ at 10 years of age in children born extremely preterm, with and without motor problems, as measured by the MABC-2, at 12 years of age.

Brain region mean ± standard deviation	Total MABC-2 ≤5th centile (n = 13)	Total MABC-2 >5th centile (n = 29)	P-value unadjusted	
Thalamus	12.8 ± 0.8	13.0 ± 0.8	0.34	
Basal ganglia	27.5 ± 1.8	28.1 ± 1.8	0.34	
Cerebellum	126.0 ± 10.3	132.9 ± 14.5	0.49	
Motor execution network	87.1 ± 5.8	88.9 ± 5.6	0.37	
Motor imagery network	140.2 ± 9.3	143.1 ± 9.0	0.34	
	**MABC-2 manual dexterity** ≤**5th centile (n = 10)**	**MABC-2 manual dexterity >5th centile (n = 32)**		
Thalamus	13.0 ± 0.9	13.0 ± 0.8	1.00	
Basal ganglia	27.9 ± 2.0	27.9 ± 1.8	0.97	
Cerebellum	130.7 ± 11.7	130.8 ± 14.3	0.53	
Motor execution network	88.4 ± 6.3	88.3 ± 5.5	0.98	
Motor imagery network	142.3 ± 10.1	142.2 ± 8.9	0.98	
	**MABC-2 Aiming and catching** ≤**5th centile (n = 13)**	**MABC-2 Aiming and catching >5th centile (n = 29)**		**P-value adjusted** *
Thalamus	12.6 ± 0.7	13.1 ± 0.8	0.052	
Basal ganglia	27.1 ± 1.5	28.3 ± 1.8	**0.04**	**0.50**
Cerebellum	120.8 ± 6.9	135.2 ± 13.6	**<0.001**	**0.02 (η** ^**2**^ ** = 0.17)**
Motor execution network	85.8 ± 4.8	89.5 ± 5.7	**0.04**	**0.48**
Motor imagery network	138.3 ± 7.8	143.9 ± 9.2	**0.049**	**0.53**
	**MABC-2 Balance** ≤**5th centile (n = 8)**	**MABC-2 Balance >5th centile (n = 34)**		
Thalamus	13.2 ± 0.6	12.9 ± 0.9	0.36	
Basal ganglia	28.3 ± 1.4	27.8 ± 1.9	0.42	
Cerebellum	126.9 ± 12.3	131.6 ± 13.9	0.43	
Motor execution network	89.7 ± 4.5	88.0 ± 5.9	0.39	
Motor imagery network	144.5 ± 7.3	141.6 ± 9.5	0.37	

*General linear model with birth weight and sex as a covariate. Significant values defined as p < 0.05. η^2^ = Partial Eta Squared.

## Discussion

This study showed the relationship between extreme prematurity, motor brain networks and regions and motor performance. There were three main findings. First, the MABC-2 results at 12 years of age showed that children born EPT had both lower component and standard scores on the MABC-2 compared with the term born control group.

Second children born EPT had significantly smaller volumes in the motor execution network, the motor imagery network, basal ganglia, thalamus and cerebellum than their term-born peers at 10 years of age. Third, children born EPT with motor impairment in aiming and catching had smaller volumes, mainly in the cerebellum, than children born EPT without motor difficulties in aiming and catching. These findings not only highlight differences in brain structure and motor performance, but also suggest potential neuroanatomical correlates of DCD in EPT children. The observed reductions in motor area volumes may reflect a structural vulnerability underlying the motor impairments characteristic of DCD.

EPT births occur at a vulnerable time of brain development and the extrauterine environment affects brain growth [6]. It is well known that prematurity leads to altered regional and global brain volumes, compared to term birth, as these have been seen on MRI scans [24,25]. In addition, it has been shown that reduced brain volumes of grey matter (d = −0.62; CI −0.48 to −0.76; p < 0.001), white matter (d = −0.53; CI −0.40 to −0.67; p < 0.001) and cerebellum (d = −0.74; CI −0.56 to −0.92; p < 0.001) persist throughout childhood in individuals born very preterm [10]. However, very few studies have explored the impact of EPT birth on the development of the brain into late childhood. Kvanta et al reported smaller white matter volumes in children born EPT compared to term-born peer at 10 years of age [26]. Some of our findings were consistent with other studies, but most studies included children born at more than 28 weeks of gestation and did not include infants born EPT. Grunewaldt et al reported that children born with an extremely low birth weight, below 1,000 grams, demonstrated reduced volumes of cerebellum and thalamus compared to term-born controls at 10 years of age. Mean volumes of the cerebellum and thalamus were 22.64 cm^3^ and 13.66 cm^3^ respectively in the preterm group, and 26.9 and 14.92 cm^3^ in the control group at 10 years of age [27]. Another study by Lax et al showed brain volumes in the thalamus were smaller in children born preterm (mean 11.3 cm^3^) compared to term-born controls at around 8 years of age. Children born preterm also had smaller basal ganglia volumes than children born at term age [28]. These studies were in line with our results. They suggest that the brain development in children born EPT with smaller volumes than term-born peers are consistent with impaired growth and that these persist in late childhood [28].

Our study found that children born EPT with motor impairments in aiming and catching had significantly reduced volumes in the areas of the brain involved in motor performance. However, differences where only seen in cerebellum when we adjusted for birth weight and sex. Catching a ball is a fundamentally complex motor skill and demonstrates skills that are crucial for executing complex movements [29]. This test requires the interaction between visual and motor systems and spatiotemporal coordinated movements before the ball is caught [30]. A review from Cameron et al demonstrated that these functions can be affected when an individual is born EPT and this could be because the developmental trajectory of the brain is altered [31]. Areas of the brain involved in motor performance have been reported to be reduced in individuals born EPT [12,27,32].

The observed association between impaired aiming and catching and reduced volumes supports the view that these structures play a crucial role in visuomotor integration. Aiming and catching require accurate visuospatial processing and coordination of eye–hand movements, which depend on functional connectivity between visual, motor execution, and motor imagery networks. The cerebellum contributes to error correction and coordination, while the basal ganglia are involved in motor learning and action selection. Our findings suggest that reduced volumes in these regions may impair the integration of visual and motor signals, leading to poorer performance in complex motor tasks such as ball handling [32].

We believe that this was the first study to explore the motor networks, basal ganglia, thalamus and cerebellum of the brains of children born EPT and motor outcomes in late childhood. Dewey et al reported smaller brain volumes in the thalamus, basal ganglia and cerebellum and motor impairment when children who were born very preterm reached seven years of age [12]. The volumes of the cerebellum and thalamus have also been shown to correlate with motor performance at 10 years of age in children born with extremely low birth weights [27]. The connectivity of the preterm brain has shown to be altered in relation to motor performance. A functional MRI study by Wheelock et al showed weaker associations between MABC-2 motor scores and connectivity between both the basal ganglia and thalamus and the motor network when children born preterm reached 12 years of age. This suggests that there is an alternative network architecture that supports motor function in children born preterm [33].

The basal ganglia and thalamus are found deep in the grey matter of the brain and they are important structures as they modulate motor performance. MRI studies have shown that the volumes of the basal ganglia and thalamus were smaller in children born very preterm when they reached term age than those born at term [34]. This may be explained by dysmaturation in the brain, due to preterm birth, since the development of these structures occur in the third trimester [34,35]. Our study found that the volume of the basal ganglia was reduced in children who were born EPT and had impaired aiming and catching. Thompson et al reported findings that were in line with our results. They found that the structural connectivity in the motor tracts involving the basal ganglia were related to motor performance when children born very preterm reached seven years of age. This demonstrated weak connectivity in the corticostriatal and thalamo-cortical tracts in very preterm children [36].

The growth of the cerebellum was the brain area that was most affected by being born EPT in this study. This is important for a wide range of motor control functions, including voluntary limb movements, cerebral-cerebellar interactions, controlling corticomotor responses, eye movements, controlling grip forces and timing [37]. The growth of the cerebellum mainly occurs during the last trimester and disturbing the normal intrauterine brain growth reduces the volume. Several causes for the underdevelopment of the cerebellum have been discussed in the literature. For example, Volpe reported that inflammation; infection, hypoxia, blood producers, steroids and undernutrition led to deficits in the cells and the cell layers. Other reasons that were discussed were disturbances in the connections with the cerebellum and cerebrum [6]. Although studies have shown neuroprotective actions that can help the brain to organize better in children born preterm. Charpak et al studied adults that were born preterm and showed that those who hade received kangaroo care until 40 weeks of age had better organization of white matter and larger volumes of cerebellum, total grey matter and basal nucei at 20 years of age [38].

Many of the children born EPT develop motor and cognitive impairments [2] that affect everyday life, including school activities, hobbies and interactions with other children. This is why interventions are so important for these children. Westendorp et al highlighted the importance of teaching aiming and catching to children with learning difficulties in an intervention study. Children who took part in the intervention practiced aiming and catching as part of a group, while the control group took part in regular physical activity classes. There were positive associations between improved aiming and catching and problem solving in the intervention group, which demonstrated that motor abilities were fundamental to their cognitive development [39].

The strengths of this study included the longitudinal design and including children born EPT and at term. The term born group was representative (with standard scores of 10–11) as a comparison group in motor performance. The limitations included the small number of children, which may have meant that some statistical differences were not detected. We acknowledge that the resulting sample size is smaller than what is typically expected in pediatric research. However, this study confronts unique challenges, including high attrition rates common in long-term follow-ups and the specific difficulties in obtaining high-quality scans from extremely preterm children, who may have more health and developmental issues affecting scan quality and participation.

Manual segmentation of the cerebellum was performed due to limitations observed with the automated segmentation methods available at the time of analysis. In particular, automated outputs frequently failed to capture the full extent of the cerebellum, with some regions missing entirely or only partially delineated. Additionally, in several cases, non-brain tissue was erroneously included in the segmentation. These inconsistencies resulted in unreliable volume estimates and, in some cases, rendered the segmentations inappropriate for analysis. To ensure anatomical accuracy and consistency across the dataset, we opted manual segmentation of the cerebellum.

While we did not conduct a power analysis due to the exploratory nature of the study and the unprecedented challenges in estimating effect sizes within this specific population, the study was carefully designed to maximize the insights that could be derived from a smaller, well-defined sample. Our approach aligns with existing literature that supports the value of qualitative and in-depth analyses in highly specialized cohorts, where the rarity and specificity of the data can offer significant contributions to the field. Including more children in the study would have been beneficial and strengthened the results. The number of children that did not participate in the follow-up study as 12 years of age was quite high, but this was to be expected due to the amount of time that had elapsed since their birth.

## Conclusions

Our study showed the long-term effects that EPT birth had on the motor networks in the brain and the impact on motor development at 12 years of age. The children born EPT demonstrated reduced brain volumes in their motor networks, thalamus, basal ganglia and cerebellum when they were compared to their term-born peers. Children born EPT who had impaired aiming and catching had a significantly smaller cerebellum, as well as reduced volume in their motor networks and basal ganglia, than children born EPT without the same motor difficulties.

## Supporting information

S1 Table Characteristics and magnetic resonance imaging findings for children born extremely preterm with, and without, motor problems at 12 years of age. (PDF)

S2 Table Characteristics and magnetic resonance imaging findings for children born extremely preterm, with, and without, motor problems in manual dexterity at 12 years of age. (PDF)

S3 Table Characteristics and magnetic resonance imaging findings for children born extremely preterm, with, and without, motor problems in aiming and catching at 12 years of age. (PDF)

S4 Table Characteristics and magnetic resonance imaging findings for children born extremely preterm, with, and without, motor problems in balance at 12 years of age. (PDF)

S1 Fig Deficiencies in automatic segmentation of the cerebellum. (PDF)
