# Supplementary material for: Brain volumes are related with motor skills at late childhood in children born extremely preterm
Source: PLoS One. 2025 Jun 13;20(6):e0326041. doi: 10.1371/journal.pone.0326041 (PMC12165354; doi:10.1371/journal.pone.0326041)
Supplement: S1 Table — (PDF) [file pone.0326041.s001.pdf]

S1 Table. Characteristics and magnetic resonance imaging findings for children born extremely preterm with, and without, motor problems at 12 years of age

	**Total MABC-2 ≤5th centile (n=13)**	**Total M-ABC-2 >5th centile (n=29)**	**P-value**
**Perinatal**			
Birth weight (grams), mean ± SD	753 ± 133	872 ± 145	**0.02**
Gestational age at birth (weeks), median (range)	25.3 (23.1-26.6)	25.6 (23.5-26.6)	0.15
Male sex, n	6	15	1.0
Small for gestational age, n	2	1	0.22
Antenatal steroids, n	11	28	0.51
Bronchopulmonary dysplasia requiring oxygen at 36 weeks, n	7	7	0.07
Intraventricular haemorrhage, grade I-II/III-IV,n	5/1	8/1	0.24
Mechanical ventilation (days), median (range)	18 (0-55)	3 (0-28)	**0.02**
Necrotizing enterocolitis Bell´s grade 2-3, n	4	3	0.17
Patent ductus arteriosus, treated with ibuprofen, n	7	19	0.73
Patent ductus arteriosus, surgical ligation, n	4	8	0.72
Retinopathy of prematurity, laser treatment, n	3	3	0.33
Sepsis, n	9	20	1.0
**Magnetic resonance imaging at term age**			
Normal/mild/moderate white matter abnormality, n	7/4/1	16/13/0	0.79
Gray matter abnormality, n	1	0	0.29
Cerebellar injury, n	1	3	0.78
**Magnetic resonance imaging at 10 years of age**			
Discrete white matter abnormality	8	14	0.51
Age at scan, median (range)	10.3 (9.1-11.3)	9.9 (9.0-11.4)	0.55
Intracranial volume, mean ± SD	1355.8 ± 89.1	1391.2 ± 88.1	0.24
Age at motor assessment (MABC-2) median (range)	12.1 (11.9-12.7)	12.2 (11.7-13)	0.38
