# Supplementary material for: Brain volumes are related with motor skills at late childhood in children born extremely preterm
Source: PLoS One. 2025 Jun 13;20(6):e0326041. doi: 10.1371/journal.pone.0326041 (PMC12165354; doi:10.1371/journal.pone.0326041)
Supplement: S2 Table — (PDF) [file pone.0326041.s002.pdf]

S2 Table. Characteristics and magnetic resonance imaging findings for children born extremely preterm, with, and without, motor problems in manual dexterity at 12 years of age

	**MABC-2 manual dexterity ≤5th centile (n=10)**	**MABC-2 manual dexterity >5th centile (n=32)**	**P-value**
**Perinatal**			
Birth weight (grams), mean ± SD	753 ± 114	861 ± 153	**0.03**
Gestational age at birth, weeks, median (range)	25.4 (23.1-26.6)	25.9 (23.5-26.6)	0.33
Male sex, n	7	14	0.28
Small for gestational age, n	2	1	0.14
Antenatal steroids, n	10	29	1.00
Bronchopulmonary dysplasia requiring oxygen at 36 weeks, n	6	8	0.06
Intraventricular haemorrhage, grade I-II/III-IV, n	3/1	10/1	0.60
Mechanical ventilation (days), median (range)	23 (0-55)	4 (0-43)	**0.01**
Necrotizing enterocolitis Bell´s grade 2-3, n	4	3	0.17
Patent ductus arteriosus, treated with ibuprofen, n	7	19	0.72
Patent ductus arteriosus, surgical ligation, n	3	9	1.00
Retinopathy of prematurity, laser treatment, n	2	4	0.62
Sepsis, n	9	20	0.23
**Magnetic resonance imaging at term age**			
Normal/mild/moderate white matter abnormality, n	5/4/1	18/13/0	0.37
Gray matter abnormality, n	1	0	0.24
Cerebellar injury, n	1	3	1.0
**Magnetic resonance imaging at 10 years of age**			
Discrete white matter abnormality	7	15	0.28
Age at scan, median (range)	9.4 (9.0-11.3)	10.0 (9.0-11.4)	0.20
Intracranial volume, mean ± SD	1374.7 ± 96.7	1382.0 ± 87.8	0.83
Age at motor assessment (MABC-2) median (range)	12.1 (12.0-12.7)	12.2 (11.7-13)	0.96
