# Supplementary material for: Brain volumes are related with motor skills at late childhood in children born extremely preterm
Source: PLoS One. 2025 Jun 13;20(6):e0326041. doi: 10.1371/journal.pone.0326041 (PMC12165354; doi:10.1371/journal.pone.0326041)
Supplement: S3 Table — (PDF) [file pone.0326041.s003.pdf]

S3 Table. Characteristics and magnetic resonance imaging findings for children born extremely preterm, with, and without, motor problems in **aiming and catching** at 12 years of age

	**MABC-2 aiming and catching ≤5th centile (n=13)**	**MABC-2 aiming and catching >5th centile (n=29)**	**P-value**
**Perinatal**			
Birth weight (grams), mean ± SD	776 ± 139	862 ± 150	0.08
Gestational age at birth, weeks, median (range)	25.6 (23.1-26.6)	25.6 (23.5-26.6)	0.39
Male sex, n	4	17	0.18
Small for gestational age, n	2	1	0.22
Antenatal steroids, n	11	28	0.51
Bronchopulmonary dysplasia requiring oxygen at 36 weeks, n	7	7	0.07
Intraventricular haemorrhage, grade I-II/III-IV, n	6/1	7/1	0.08
Mechanical ventilation (days), median (range)	14 (0-55)	3 (0-41)	0.15
Necrotizing enterocolitis Bell´s grade 2-3, n	4	3	0.17
Patent ductus arteriosus, treated with ibuprofen, n	5	21	0.08
Patent ductus arteriosus, surgical ligation, n	4	8	0.72
Retinopathy of prematurity, laser treatment, n	2	4	1.00
Sepsis, n	10	19	0.45
**Magnetic resonance imaging at term age**			
Normal/mild/moderate white matter abnormality,n	5/6/1	18/11/0	0.13
Gray matter abnormality, n	1	0	0.29
Cerebellar injury, n	3	1	0.07
**Magnetic resonance imaging at 10 years of age**			
Discrete white matter abnormality	7	15	1.00
Age at scan, median (range)	10.4 (9.1-11.3)	9.8 (9.0-11.4)	0.59
Intracranial volume, mean ± SD	1340.4 ± 82.3	1398.0 ± 87.1	0.05
Age at motor assessment (MABC-2) median (range)	12.1 (12.0-12.7)	12.2 (11.7-13)	0.53
