# Supplementary material for: Brain volumes are related with motor skills at late childhood in children born extremely preterm
Source: PLoS One. 2025 Jun 13;20(6):e0326041. doi: 10.1371/journal.pone.0326041 (PMC12165354; doi:10.1371/journal.pone.0326041)
Supplement: S4 Table — (PDF) [file pone.0326041.s004.pdf]

S4 Table. Characteristics and magnetic resonance imaging findings for children born extremely preterm, with, and without, motor problems in balance at 12 years of age

	MABC-2 Balance ≤5th centile (n=8)	MABC-2 Balance >5th centile (n=34)	P-value
**Perinatal**			
Birth weight (gram), mean ± SD	804 ± 137	843 ± 155	0.50
Gestational age at birth, weeks, median (range)	25.8 (23.1-26.6)	25.6 (23.5-26.6)	0.91
Male sex, n	4	17	1.00
Small for gestational age, n	1	2	0.48
Antenatal steroids, n	8	31	1.00
Bronchopulmonary dysplasia requiring oxygen at 36 weeks, n	3	11	1.00
Intraventricular haemorrhage, grade I-II/III-IV,n	4/1	9/1	0.07
Mechanical ventilation (days), median (range)	12 (0-55)	4 (0-43)	0.59
Necrotizing enterocolitis Bell´s grade 2-3, n	2	5	0.61
Patent ductus arteriosus, treated with ibuprofen, n	3	23	0.12
Patent ductus arteriosus, surgical ligation, n	2	10	1.00
Retinopathy of prematurity, laser treatement, n	0	6	0.32
Sepsis, n	5	24	0.67
**Magnetic resonance imaging at term age**			
Normal/mild/moderate white matter abnormality,n	4/4/0	19/13/1	0.84
Gray matter abnormality, n	0	1	1.0
Cerebellar injury, n	1	3	1.0
**Magnetic resonance imaging at 10 years of age**			
Discrete white matter abnormality	5	17	0.70
Age at scan, median (range)	9.4 (9.1-10.8)	10.3 (9.0-11.4)	0.07
Intracranial volume, mean ± SD	1386.5 ± 80.5	1378.8 ± 91.8	0.82
Age at motor assessment (MABC-2) median (range)	12.0 (11.9-12.7)	12.2 (11.7-13)	0.23
