# Supplementary figures and images for: Brain volumes are related with motor skills at late childhood in children born extremely preterm
Source: PLoS One. 2025 Jun 13;20(6):e0326041. doi: 10.1371/journal.pone.0326041 (PMC12165354; doi:10.1371/journal.pone.0326041)

**Supplementary figure 1. Deficiencies in automatic segmentation of the cerebellum**

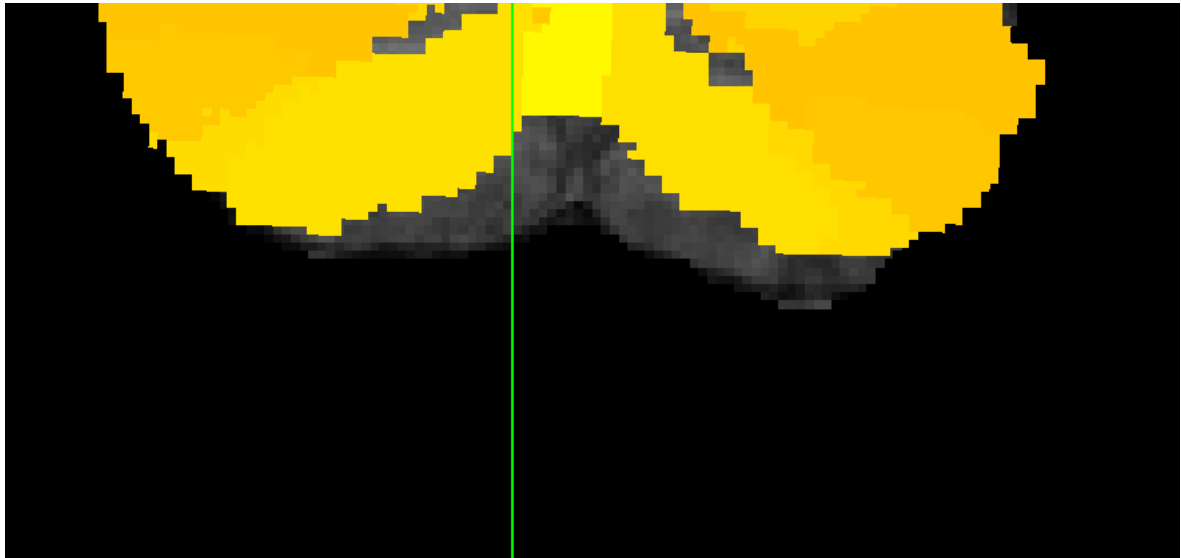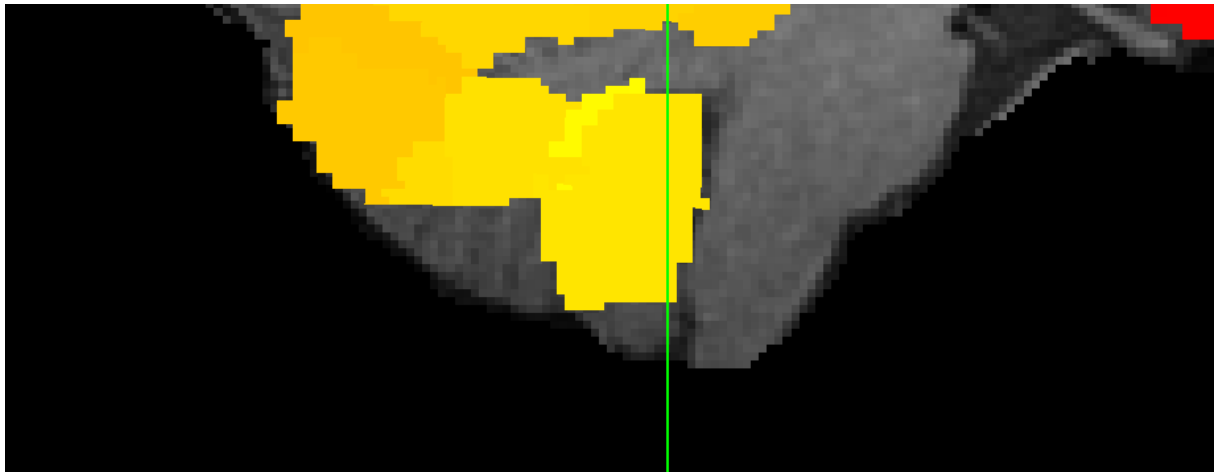

Supplement: S1 Fig — (PDF) [file pone.0326041.s005.pdf]
